# Seasonal Dynamics of Resource Availability and Human Presence Shape Habitat Use of Large Carnivores in Chad

**DOI:** 10.1002/ece3.73957

**Published:** 2026-07-13

**Authors:** Chiara Fraticelli, Ali Shams, Stijn Verschueren, Ousmane Ourde, Baudin Fekoua, Abdoulaye Abakar Zayed, Herwig Leirs, Hans Bauer

**Affiliations:** ^1^ Evolutionary Ecology Group, Department of Biology University of Antwerp Antwerp Belgium; ^2^ African Parks Network, Greater Zakouma Ecosystem Zakouma Chad; ^3^ Institute for Communities and Wildlife in Africa (iCWild) University of Cape Town Cape Town South Africa; ^4^ Conservation Biology Division University of Bern Bern Switzerland; ^5^ Cheetah Conservation Fund Otjiwarongo Namibia; ^6^ Wildlife Conservation Research Unit, Biology Department University of Oxford Oxford UK

**Keywords:** camera trap, coexistence, large carnivores, occupancy, pastoralism, seasonality

## Abstract

Understanding how seasonal changes in resources and human activity influence large carnivore space use is critical for effective conservation, yet such information is scarce for Central African savanna ecosystems. This study used occupancy modelling with data from camera traps deployed during the dry and wet seasons in Zakouma National Park (NP), Chad, to assess seasonal patterns of large carnivore occurrence and their environmental drivers. Five large carnivore species were detected: lion, leopard, cheetah, spotted hyaena and striped hyaena. Prey availability emerged as the primary driver of carnivore space use in the dry season. Detections declined markedly in the wet season; occupancy remained similar across seasons for lions and leopards, whereas other species exhibited reduced occupancy in the wet season. Our results indicate that Zakouma NP functions as a critical refuge during the dry season, when anthropogenic pressure intensifies in community lands due to the movement of transhumant pastoralists. During the wet season, reduced human presence in the broader landscape, combined with decreasing prey availability and flooding that renders parts of Zakouma NP unsuitable, allows several species to expand their range beyond the park. Within the Greater Zakouma Ecosystem, these seasonal dynamics highlight the importance of landscape‐level conservation approaches that account for seasonal shifts in resource availability and human land use to support large carnivore persistence.

## Graphical abstract

Seasonal variation in resource availability and human activity can strongly influence large carnivore space use, yet these dynamics remain poorly documented in Central African savannas. Using dry and wet season camera‐trap surveys in Zakouma National Park, Chad, we show that prey availability drives carnivore space use in the dry season, and that detections decline in the wet season, with stable occupancy for lions and leopards but reduced occupancy for other species. These findings demonstrate the role of protected areas as seasonal refugia and highlight the importance of landscape‐level conservation strategies that account for shifting resource distribution and transhumant land use.

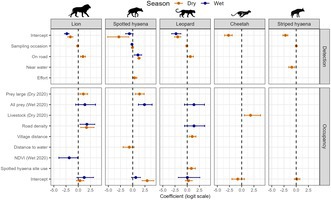

## RÉSUMÉ

La compréhension des effets des changements saisonniers des ressources et de l'activité humaine sur l'utilisation de l'espace par les grands carnivores est. essentielle pour une conservation efficace, mais ces informations sont rares pour les écosystèmes de savane d'Afrique centrale. Cette étude a utilisé des pièges photographiques déployés pendant les saisons sèche et des pluies dans le parc national de Zakouma, au Tchad, afin d'évaluer les schémas saisonniers de présence des grands carnivores et les facteurs environnementaux qui les influencent. Cinq espèces de grands carnivores ont été détectées: le lion, le léopard, le guépard, la hyène tachetée et la hyène rayée. La disponibilité des proies est. apparue comme le principal facteur déterminant l'utilisation de l'espace par les carnivores pendant la saison sèche. Les détections ont nettement diminué pendant la saison des pluies; l'occupation est. restée similaire d'une saison à l'autre pour les lions et les léopards, tandis que les autres espèces ont montré une occupation réduite pendant la saison des pluies. Nos résultats indiquent que le parc national de Zakouma fonctionne comme un refuge essentiel pendant la saison sèche, lorsque la pression anthropique s'intensifie dans les zones communautaires en raison des déplacements des éleveurs transhumants. Pendant la saison des pluies, la présence humaine réduite dans la région, combinée à la diminution de la disponibilité des proies et aux inondations qui rendent certaines parties du parc national de Zakouma impraticables, permet à plusieurs espèces d'étendre leur aire de répartition au‐delà du parc. Au sein du grand écosystème de Zakouma, ces dynamiques saisonnières soulignent l'importance des approches de conservation à l'échelle du paysage qui tiennent compte des variations saisonnières de la disponibilité des ressources et de l'utilisation des terres par l'homme afin de favoriser la persistance des grands carnivores.

## 1 Introduction

Large African carnivores have important ecological roles and influence the structure of the ecosystems they inhabit (Ripple et al. 2014; Wolf and Ripple 2017). At the same time, they are more vulnerable to threats due to their slow life histories, low population densities, wide home ranges and propensity for conflict with livestock (Wolf and Ripple 2017). Predator distributions are commonly shaped by prey availability and catchability, which is in turn shaped by water availability and habitat type and can vary cyclically, especially in environments with strong seasonal dynamics (Loveridge et al. 2009; Valeix et al. 2010). Competition and risk avoidance, both with humans or with other sympatric large carnivores, can also influence space use, both spatially and temporally (Oriol‐Cotterill, Macdonald, et al. 2015; Oriol‐Cotterill, Valeix, et al. 2015; Say‐Sallaz et al. 2025; Verschueren et al. 2024).

Well‐managed Protected Areas (PA) provide the backbone for large carnivore conservation, but populations in open systems may face threats beyond park boundaries that negatively affect their viability through source‐sink dynamics (Bauer et al. 2022). Understanding the factors influencing species' habitat use is essential to guide conservation efforts. For example, several studies have shown the importance of water availability as a key driver of prey dispersion and predictability, which in turn influences carnivore space use (Loveridge et al. 2009; Valeix et al. 2010). In semi‐arid savanna systems, seasonal rainfall reconfigures water distribution across the landscape, where prey tend to aggregate at scarce water points during the dry season and disperse more widely during the wet season (Fraticelli et al. 2025; Laizer et al. 2014; Loveridge et al. 2009; Valeix 2011). Vegetation type and structure further mediate prey encounter rates and hunting efficiency, with species‐specific habitat preferences and distinct hunting modes (Atkinson et al. 2022; Hopcraft et al. 2005).

Simultaneously, carnivores actively avoid anthropogenic risks, modifying their behaviour and distribution in response to human factors (Carter et al. 2017; Loveridge et al. 2017), but avoidance and tolerance of anthropogenic disturbance can also vary within the carnivore guild and depending on the local context. Areas with anthropogenic disturbance, for example, can act as a refuge for subordinate carnivores from more dominant competitors that have a lower level of tolerance (Abrahms et al. 2025). Seasonality also has an influence on carnivores' tolerance of human activities and many areas present seasonal variation in livestock depredation, with large carnivores switching to prey on livestock in seasons when wild prey density is lower or when livestock is herded farther from settlements and into habitats where they are more vulnerable to predation (Kuiper et al. 2015; Loveridge et al. 2017; Mukeka et al. 2019). As a result, understanding how large carnivores navigate seasonal landscapes is critical for designing protected area networks that remain functional year‐round, especially in unfenced systems where animals move across park boundaries (Bauer et al. 2022).

Intraguild interactions can also shape habitat use and influence population densities of sympatric carnivores. Negative relationships between the population densities of dominant and subordinate competitors have been observed for numerous carnivore species, including wolves ( *Canis lupus* ) and coyotes ( *Canis latrans* ; Berger and Gese 2007), and lions ( *Panthera leo* ) and wild dogs ( *Lycaon pictus* ) (Swanson et al. 2014). In contrast, other subordinate species seem largely unaffected by intraguild competitors, such as leopards ( *Panthera pardus* ; Rafiq et al. 2020) or cheetahs ( *Acinonyx jubatus* ; Swanson et al. 2014) in the presence of lions, where fine‐scale response in time and space promotes co‐existence. Scavenging and kleptoparasitism further structure carnivore guild interactions (Amorós et al. 2020; Balme, Miller, et al. 2017; Bashant et al. 2020; Périquet, Fritz, and Revilla 2015; Périquet, Valeix, et al. 2015). Overall, intraguild interactions and their effects on subordinate species are highly context dependent (Greco et al. 2021; Haswell et al. 2017) and require an area‐specific understanding.

Across Central Africa, PAs are experiencing progressive degradation (Scholte et al. 2022), and although large carnivores continue to persist within these systems, their populations show marked declines (Brugière et al. 2015; Shams et al. 2024). Zakouma National Park (NP) in Chad is considered a rare success story for PAs in Central Africa (Scholte et al. 2022), with a significant increase in herbivore numbers in the last 15 years (Fraticelli et al. 2021). Carnivores are more difficult to monitor and less research has been carried out on these species in the region, which makes it harder to understand the population trends. Abundance of lions and spotted hyaenas in the park was estimated in 2005 (Vanherle 2011), and then only lion population size was estimated again in 2020 (Fraticelli et al. in review) with no significant changes in population size detected. Cheetah were only estimated in 2023 (Shams et al. 2024), while leopards and striped hyaenas in the area were never studied. Nevertheless, all five of these large carnivores are commonly observed in the park.

Zakouma NP is the core protected area of the Greater Zakouma Ecosystem (GZE), which also includes Siniaka Minia NP and Bahr Salamat Faunal Reserve. The GZE is one of only two ecosystems classified as functional conservation areas in Central Africa (Brugière et al. 2015). Located within a region that retains high landscape‐level integrity and relatively low human impact from land use change (Grantham et al. 2020; Visser et al. 2025), southern Chad and the adjacent regions of the Central African Republic (CAR) have the potential to become the largest interconnected conservation area in Central Africa.

Outside of the NPs, the GZE holds both permanent human settlement and nomadic herders (i.e., transhumants) with large herds of cattle, camels and small ruminants who spend the wet season in the north of Chad and the dry season in CAR or southern Chad, including the outskirts to the NPs. PAs in the GZE are unfenced and wildlife regularly moves between the NPs and the surrounding human landscape (Dolmia et al. 2007; Labuschagne 2014; Clark et al. 2023; Fraticelli et al. 2025, 2026). This is especially evident in the wet season when up to 30% of Zakouma NP is flooded: migratory antelopes such as hartebeest ( *Alcelaphus buselaphus* ) and tiang ( *Damaliscus lunatus* ) travel up to 180 km north of the park (Poilecot 2006), but other species such as elephants (Dolmia et al. 2007; Labuschagne 2014), giraffes (Clark et al. 2023) and lions (Fraticelli et al. 2025) also range significanlty more outside of Zakouma NP, although not as far. While for other large carnivore species there is no tracking data to show the extent of movement inside or outside of Zakouma NP, reports of human carnivore conflict seem to indicate similar seasonal patters for leopards, while spotted hyaenas seem to be permanently present throughout the wider ecosystem (Fraticelli et al. 2026). In the wider landscape, carnivores are at risk of anthropogenic mortality ranging from watehole poisoning to targeted retaliatory killing (Fraticelli et al. 2026). There is variation in attitudes towards carnivores depending on the livelihood, with tranhumanst being more likely to want to retaliate against depredation, especially when the stock killed was the more valuable cattle (Fraticelli et al. 2026), which is similar to what is observed with Maasai pastoralists in Kenya (Ontiri et al. 2019).

Thanks to the extreme seasonal variations in water availability and anthropogenic pressures, the GZE presents a prime opportunity to investigate the drivers behind these seasonal dynamics in large carnivore movement. We conducted the first extensive camera trap survey in Zakouma NP in 2020 over both the dry and wet season to document the presence, distribution and seasonal differences in space use for the five large carnivore species present. We used occupancy modelling, which accounts for imperfect detection (MacKenzie et al. 2002), to explore the environmental and anthropogenic drivers influencing predator space use in both seasons. We predict that seasonal changes alter habitat use by large carnivores. We discuss the results in the context of seasonal and landscape‐level patterns of habitat use and conservation planning and provide baseline information on the presence and habitat requirements of large carnivores in a regionally important conservation area.

## 2 Methods

### 2.1 Study Area

**FIGURE 1:**
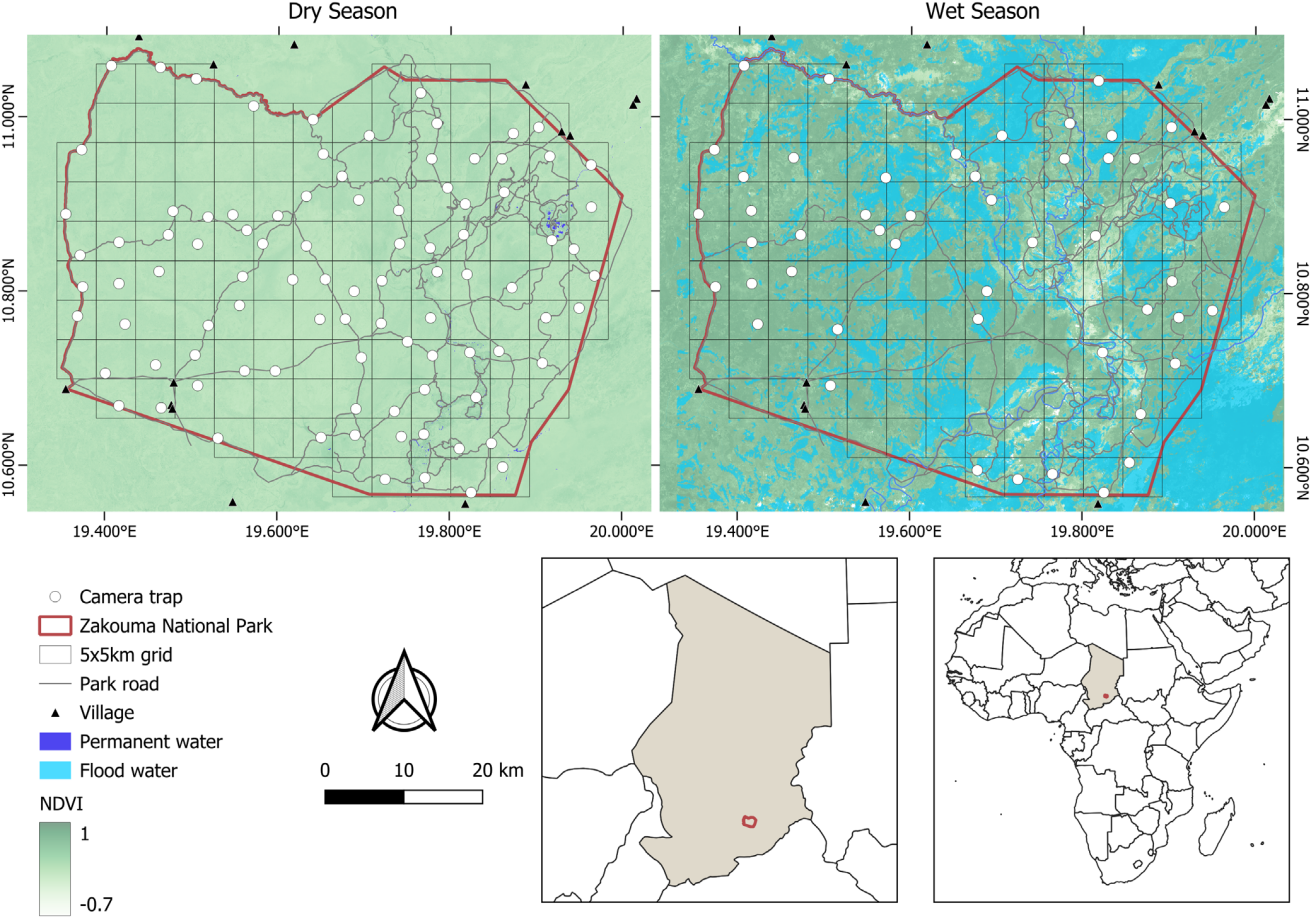
Dry season and wet season maps of Zakouma National Park showing the placement of the cameras included in the analysis, as well as some of the features included as covariates.

Zakouma NP (Figure 1) is a 3054km^2^ protected area at the core of the GZE in the south east of Chad (19.350, 10.568; 19.999, 11.077). Created in 1963, it is now considered one of the most successful PAs in Central Africa, with an exponential increase in herbivore species and ongoing recovery of the elephant population (Fraticelli et al. 2021), thanks to the commitment of the Government of Chad and the long term financial support of the European Union (Scholte et al. 2018), as well as the management improvements brought by the delegate management to African Parks Network (Scholte et al. 2022).

Located in the Sudano‐sahelian ecosystem, Zakouma NP receives an average 800 mm of rain in the months between June and October. Usually starting from July, parts of the park, especially the eastern side, flood due to several large seasonal rivers flowing into Zakouma NP from the periphery. A cold dry season going from October to February is followed by a hot dry season, when surface water is only present in a few permanent pans in the east. The eastern half of Zakouma NP, where wildlife concentrates in the dry season, has more developed infrastructure, including more roads, permanent and temporary camps, and a higher concentration of rangers, and receives tourists from December to May.

Five large carnivores are present: lion, leopard, spotted hyaena, cheetah and striped hyaena. Other wildlife includes elephant ( *Loxodonta africana* ), buffalo ( *Syncerus caffer* ), giraffe ( *Giraffa camelopardalis* ), roan antelope ( *Hippotragus equinus* ), hartebeest, tiang, waterbuck ( *Kobus ellipsiprymnus* ) and olive baboon ( *Papio anubis* ), as well as a range of smaller herbivores and carnivores. Wildlife species adapt to the wet season conditions by changing their range, with species such as hartebeest and tiang migrating up to 180 km north of the NP (African Parks, unpublished data; Poilecot 2006), elephants dispersing west in the corridor towards Siniaka Minia NP (Dolmia et al. 2007; Labuschagne 2014), while other species such as giraffe, roan and lions move to different areas inside the park or in the vicinity (Clark et al. 2023; Fraticelli et al. 2025).

Although Zakouma NP benefits from effective protection, the surrounding areas support permanent settlements and are traversed by transhumant pastoralists who move large herds of livestock between northern Chad in the wet season and southern Chad or the CAR in the dry season. During the dry season, livestock and herders concentrate around the park boundaries, increasing anthropogenic pressure and occasionally entering the park. Because protected areas in the GZE are unfenced, wildlife regularly moves between the park and adjacent community lands, resulting in varying levels of interaction between wildlife, livestock and human activities throughout the year (Dolmia et al. 2007; Labuschagne 2014; Clark et al. 2023; Fraticelli et al. 2025, 2026).

### 2.2 Data Collection

A 5 × 5 km grid was overlaid on Zakouma NP (Figure 1), based on the smallest home range of large carnivores (Asfaw et al. 2025). Grid cells that were not accessible were excluded from the survey. During the wet season survey, additional grid cells were excluded due to floods, and some cameras were moved within the grid cells to areas with lower flood risk. Some areas, such as the north‐west off‐road section of the park, only became accessible at the end of the dry season after the bushfires reduced vegetation cover; thus, camera deployment in this area was only possible for the wet season.

One camera was deployed in each of the grid cells, in locations that would maximise detection of the wildlife; these included active game trails, near waterholes and unpaved roads. The cameras were deployed at a height of 40‐50 cm from the ground. The majority of the camera traps were attached to trees; where trees were not available or not suited for camera attachment, a sand coloured metal stake was hammered into the ground and the camera attached to it. We used three different camera models: Cuddeback Xchange (Cuddeback, Green Bay, Wisconsin, USA), Bushnell CORE, Bushnell Trophy Cam HD Aggressor (Bushnell Outdoor Products, Overland Park, Kansas, USA), with flash being white, infrared or covert. Cameras with infrared or covert flash were used along the border roads of the park and in areas of high human presence to minimise interference and theft by people, while white flash was used in less disturbed areas to improve species identification. Different cameras were set with equivalent settings and no delay between triggers, but cameras with white flash at night have a longer recharging time after the flash is triggered.

Cameras were deployed in 120 grid cells throughout Zakouma NP during the survey year. Out of these, we removed from the database the cameras that were stolen, damaged by bush fires or floods, or malfunctioned and only kept stations where we had at least two full sampling occasions of data in the survey period (10 consecutive days).

### 2.3 Data Analysis

Species identification was done manually for the dry season survey, and all images of large carnivores were double checked by a second person to avoid misidentifications. The images of the 2020 wet season were identified with the help of TrapTagger (WildEye 2023), an open source web application that combines use of AI and manual annotations, with the Sub‐Saharan Africa Species Classifier (version 1.0.0) which was trained, among others, on the images of the Zakouma NP 2020 dry season survey. All images of key species were also further verified manually.

To respect the assumption of closure (MacKenzie et al. 2006), each sampling period was 90 days, with the dry season survey running from 15th of January to 13th of April and the wet season survey from the 1st of July to 28th of September. Observations were grouped into sampling occasions of 5 days, thus totalling 18 sampling occasions per session. The home range of some of these species (Fraticelli et al. 2025) is significantly larger than the sampling units and violates occupancy assumptions (MacKenzie et al. 2006), thus we interpret the output as intensity of site use rather than true occupancy (Mills et al. 2020; Reasoner et al. 2024; Suraci et al. 2021).

We extracted the number of independent detections per species, assuming a delay of 60 min between detections of the same species for them to be independent (e.g., Bahaa‐el‐din et al. 2016; Tobler et al. 2008). We calculated the relative abundance index (RAI), or the number of independent detections/number of trap‐nights × 100; and the naïve occupancy, or the proportion of active stations that detected the target species at least once.

Covariates were selected to represent resource availability, environmental characteristics and anthropogenic factors that have an influence on carnivore space use (Table 1). Prey availability was included because prey distribution is a primary driver of carnivore density and distribution (Carbone et al. 2011; Loveridge et al. 2009; Van Orsdol et al. 1985). We considered the log‐transformed RAI of wild prey to be an index of prey availability for each station. Prey species were separated into large prey of > 250 kg: giraffe, buffalo and roan antelope; medium prey between 70 kg and 250 kg: waterbuck, greater kudu ( *Tragelaphus strepsiceros* ), hartebeest and tiang; and small prey < 70 kg: kob ( *Kobus kob* ), warthog ( *Phacochoerus africanus* ), bushbuck ( *Tragelaphus scriptus* ), reedbuck ( *Redunca redunca* ), red fronted gazelle ( *Eudorcas rufifrons* ), oribi ( *Ourebia ourebi* ) and common duiker ( *Sylvicapra grimmia* ).

**TABLE 1:** Covariates included in the occupancy models for the five large carnivores in the dry and wet season. Flash type: White flash (W) and dark flash (D) including covert flash (CV) and infra‐red flash (IR).

Covariate	Code	Source	Unit	Dry season	Wet season
**Detection**					
Flash type	Fla	Survey metadata	Sample size	W = 69 D = 24 (23 CV +1 IR)	W = 29 D = 20 (17 CV + 3 IR)
Effort	Eff	Survey	Number of camera nights during 5 days sampling period	Mean = 4.74 Range = 0–5	Mean = 2.29 Range = 0–5
Water presence at deployment location	locW	Survey metadata		Yes = 41 No = 52	*not included in analysis
Deployment on unpaved road	locR	Survey metadata		Yes = 55 No = 38	Yes = 28 No = 21
Sampling occasion	So	Survey metadata		1–18	1–18
**Occupancy**					
*Prey*					
Large prey (> 250 kg)	Pla	Survey	Detections/100 trap nights	Mean = 23 Range = 0–130	Mean = 24 Range = 0–136
Medium prey (70 kg–250 kg)	Pmed	Survey	Detections/100 trap nights	Mean = 10 Range = 0–94	Mean = 11 Range = 0–94
Small prey (< 70 kg)	Psm	Survey	Detections/100 trap nights	Mean = 40 Range = 0–397	Mean = 37 Range = 0–345
All prey species	Pall	Survey	Detections/100 trap nights	Mean = 73 Range = 0–473	Mean = 72 Range = 0–497
*Environmental variables*					
Distance to surface water (dry season)	Wdry	Smallest Euclidian distance from a station to the closest surface water in dry season	km	Mean = 8.95 Range = 0.06–26.79	*Not included in analysis
Surface water in sampling unit (wet season)	Wflo	Surface area covered in water in each grid cell	km^2^	*not included in analysis	Mean = 7.23 Range = 0.39–18.95
NDVI	NDVI	Average calculated with Google Earth Engine over each survey period		Mean = 0.25 Range = 0.18–0.33	Mean = 0.63 Range = 0.34–0.75
*Anthropogenic variables*					
Livestock	Stock	Survey	Detections/100 trap nights	Mean = 2.32 Range = 0–158	Mean = 0.1 Range = 0–6 *not included in analysis
Distance to park boundary	Park	Smallest Euclidian distance from a station to the park boundary	km	Mean = 9.21 Range = 0–22.45	Mean = 7.45 Range = 0–22.45
Distance to closest village	Villa	Smallest Euclidian distance from a station to the closest centre of village	km	Mean = 14.36 Range = 1.34–29.41	Mean = 14.21 Range = 1.59–25.67
Density of park roads in sampling unit	Road	Km of roads in the sampling unit calculated from GPS tracks of roads in the park	km	Mean = 8.34 Range = 0–37.79	Mean = 7.21 Range = 0–25.15
*Predator intensity of site use*					
Lion intensity of site use	Lion	Resulting from species models	Ψ	Mean = 0.61 Range = 0.01–0.96	Mean = 0.63 Range = 0.02–0.99
Spotted hyaena intensity of site use	Sphy	Resulting from species models	Ψ	Mean = 0.87 Range = 0.40–0.99	Mean = 0.62 Range = 0.01–0.99

Water availability is represented in the dry season by distance to permanent water and in the wet season by extent of seasonal surface water. These variables were included to account for the influence of water availability and flooding on habitat suitability, prey distribution and animal movement (Valeix et al. 2010). Normalised Difference Vegetation Index (NDVI) was used as an indicator of habitat structure and primary productivity, which can influence both prey distribution and hunting opportunities (Bauer et al. 2025; Rich et al. 2017). We used Google Earth Engine (Gorelick et al. 2017) to calculate the distance from surface water (Pekel et al. 2016) in the dry season and NDVI in the wet and dry season with Sentinel‐2 satellite images with a 10 m resolution, calculating the mean values for each grid square over each survey period. Wet season surface water was extracted from a ground‐truthed spatial layer of flooded areas provided by the park management (African Parks).

As anthropogenic variables we included livestock availability, which represents both anthropogenic presence and a potential alternative food source; distance to park boundary and villages as proxy to human presence and potential edge effect (Loveridge et al. 2017; Wilkinson, Xu, et al. 2024); and density of park roads, which is both a proxy for human disturbance and for anti‐poaching effort (Patterson et al. 2025; Schooler et al. 2022). Livestock presence was included as RAI, but due to the presence of an extremely high outlier, we capped livestock at the 99th percentile. Distance to park boundary, distance to villages and density of park roads were calculated from up‐to‐date spatial files provided by the park management (African Parks).

The continuous variables were *z*‐standardised (*x* − *x*/*σ*) to a mean 0 and unit standard deviation. We checked correlation between covariates with Pearson's correlation test; if two covariates were strongly correlated (|*r*| > 0.7), we only included in the final model the covariate with the best fit.

We fitted separate single‐season, single‐species occupancy models to compare predictors of both occupancy (*ψ*) and detection probability (*p*) for five large carnivores (lion, spotted hyaena, leopard, cheetah and striped hyaena) in the dry and in the wet season using the ‘spOccupancy’ package (Doser et al. 2022) in R (ver. 4.4.2; R Core Team 2024). Seasons were analysed independently because our objective was to assess species‐specific patterns of space use and covariate associations under the two different seasonal ecological conditions rather than estimate occupancy dynamics between seasons. Although multi‐species occupancy models can improve estimation for rare species by borrowing information across taxa, our focus was on species‐specific responses to environmental and anthropogenic covariates. A single‐species occupancy model provides direct estimates for each species, without imposing a shared community response structure which would be influenced by the substantial variation in detections between species in our study. We ran all models with three parallel chains of 15,000 Markov Chain Monte Carlo (MCMC) iterations each, discarding 5000 iterations during the burn‐in phase and with a thinning interval of 5. Model convergence was diagnosed using the Gelman‐Rubin statistic (R̂) and visual inspection of trace plots. We compared and ranked the models based on the Watanabe‐Akaike information criterion (WAIC).

We first modelled detection, keeping occupancy constant, with covariates thought to influence detection (Table 1). As potential detection covariates we included flash type (white flash and dark flash, the latter including infra‐red flash and covert flash), effort (number of trap nights in a 5‐day sampling period) to account for unequal sampling effort, presence of water in the immediate vicinity of the camera (dry season only) and whether the camera was deployed on an unpaved road or not on the road. We considered models within 2 ΔWAIC units of each other to have comparable empirical support, and modelled these covariates combined to identify the top detection model.

We then used the top detection model for each species to model occupancy. These were tested in four groups of univariate models (Table 1): relative abundance of prey, environmental variables, anthropogenic variables and dominant predator site use intensity. Predator site use covariates were not included for lions and only lion occupancy was included for spotted hyaenas, while occupancy of both of these species was included in the models of the three subordinate predators.

For the final model we combined the top covariates from each of the four groups and selected the top model (ΔWAIC = 0). We assessed fit of the final models by comparing observed data to simulated data using Freeman‐Tukey discrepancy and computed Bayesian *p*‐value as a summary of posterior predictive check across sites and across replicates (Kéry and Royle 2016). A *p*‐value > 0.1 and < 0.9 was considered as an adequate fit (Hobbs and Hooten 2015). Where the models did not show an adequate fit we also plotted the difference in the discrepancy measure between the replicate and actual data across each of the sites to identify sites whose discrepancy measure for the actual data is much larger than the corresponding discrepancy for the replicate data. If appropriate, we removed outliers before building the model following the same approach as above. Alternatively, we grouped data into 10‐days sampling occasions instead of 5‐days before building the model following the same approach as above.

We finally plotted the predictions based on the final model for each species and each season, keeping the non‐target covariates constant at 0.

## 3 Results

Our final dataset for analysis included 93 stations for the dry season and 49 stations for the wet season. The dry season resulted in 7933 trap nights (85 ± 13 per station), while the wet season resulted in 2022 trap nights (41 ± 29 per station). The lower number of stations and trap nights in the wet season was due to the inaccessibility of most areas during this season, causing many cameras to exhaust batteries or memory space, or have no visibility due to the tall grass before the end of the survey period.

All species had fewer detections and lower naïve occupancy in the wet season compared to the dry season (Table 2), with lion and spotted hyaenas showing a similar decrease (38% and 35% lower naïve occupancy, 32% and 31% lower RAI, respectively), leopards more than halving both naïve occupancy (64% decrease) and RAI (55% decrease), striped hyaena detected a single time in the wet season (95% decrease in naïve occupancy and 97% decrease in RAI), and no cheetah detected in the wet season.

**TABLE 2:** Results of the detections of the five large carnivores in the two seasons.

Species	Lion		Spotted hyaena		Leopard		Cheetah		Striped hyaena	
Season	Dry	Wet	Dry	Wet	Dry	Wet	Dry	Wet	Dry	Wet
Independent detections (60 min)	157	27	753	133	104	12	14	0	119	1
Stations with detections	49	16	79	27	42	8	12	0	40	1
Naïve occupancy	0.53	0.33	0.85	0.55	0.45	0.16	0.13	0	0.43	0.02
RAI	1.98	1.34	9.49	6.58	1.31	0.59	0.18	0	1.5	0.05

Abbreviation: RAI, relative abundance index.

All parameters in our final models achieved convergence (R̂ < 1.1). The posterior predictive checks of the top models for spotted hyaena and striped hyaena in the dry season resulted in a Bayesian *p*‐value across sites of < 0.1, and for cheetah in the dry season and leopard in the wet season resulted in a Bayesian *p*‐value across replicates of > 0.9 indicating poor model fit (Figure S1). For striped hyaena, plotting the difference in the discrepancy measure between the replicate and actual data across each of the sites showed a single outlier. Removing it from the analysis resulted in Bayesian *p*‐values within the acceptable range and this was kept as the final model (Table 3, Figure S1). For cheetah and leopard, where detections were low, grouping the data in 10‐days instead of 5‐days sampling occasions resulted in improved model fit (Table 3, Figure S1). For spotted hyaena none of the approaches we tried improved model fit. We report the results of the models including all sites and using 5‐days sampling occasion, but the results of this model need to be interpreted with care.

**TABLE 3:** Model selection for occupancy (*Ψ*) and detection (*p*) probabilities of five large carnivores in the dry and wet season. *K* represents the number of estimated parameters; *p* is the Bayesian *p*‐value (*p* sites: Across sites; and *p* rep: Across replicates). Final model selected is shown in bold. This table includes only top‐ranked models (ΔWAIC < 2.00), for all models see Table S1. For covariate definitions, refer to Table 1.

Dry season						Wet season					
Models	*k*	WAIC	ΔWAIC	*p* sites	*p* rep	Models	*k*	WAIC	ΔWAIC	*p* sites	*p* rep
*Lion*											
*Ψ*(Pla + Road) *ρ*(locR + so)	6	781.81	0.00	0.12	0.50	*Ψ*(Pall + Road + NDVI) *ρ*(.)	5	162.88	0.00	0.42	0.25
*Spotted hyaena*											
***Ψ*(Pla + Wdry) *ρ*(locR + so + eff)**	**7**	**1723.48**	**0.00**	**0.00**	**0.61**	***Ψ*(Pall) *ρ*(so + locR)**	**5**	**310.08**	**0.00**	**0.36**	**0.69**
*Ψ*(Pla) *ρ*(locR+so+eff)	6	1723.84	0.37	0.00	0.61	*Ψ*(Pall+lion) *ρ*(so+locR)	6	311.65	1.57	0.36	0.69
*Ψ*(Pla + Wdry+lion) *ρ*(locR+so+eff)	8	1725.05	1.58	0.00	0.62						
*Leopard*						*(10‐day sampling occasion)*					
***Ψ*(Villa + sphy) *ρ*(so + locR)**	**6**	**642.07**	**0.00**	**0.12**	**0.70**	***Ψ*(Pall + Road) *ρ*(.)**	**4**	**73.39**	**0.00**	**0.46**	**0.62**
*Ψ*(Villa+Park+sphy) *ρ*(so+locR)	7	643.85	1.78	0.10	0.69	*Ψ*(Road) *ρ*(.)	3	74.60	1.21	0.41	0.62
						*Ψ*(Pall) *ρ*(.)	3	75.38	1.99	0.44	0.61
*Cheetah (10‐day sampling occasion)*											
***Ψ*(stock) *ρ*(.)**	**3**	**133.76**	**0.00**	**0.64**	**0.78**						
*Striped hyaena (excluding outlier)*											
***Ψ*(.) *ρ*(so + locW)**	**4**	**658.57**	**0.00**	**0.19**	**0.36**						

**FIGURE 2:**
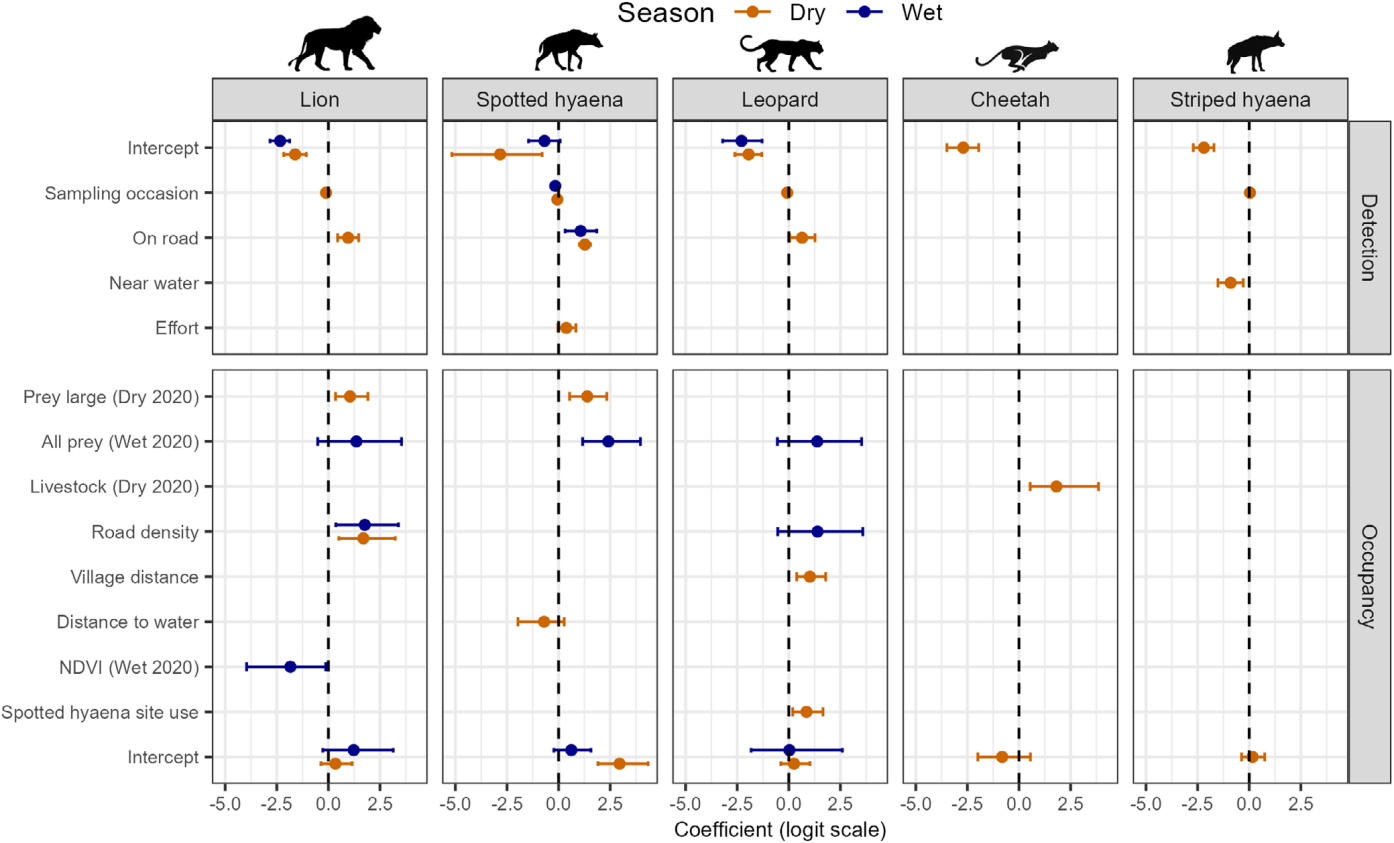
Coefficients on the logic scale with 95% CI for the selected model for each species and season.

Lions showed higher occupancy in the wet season, although with high overlap between credible intervals (CI), while their detection probability was lower in the wet season (Table 4). Spotted hyaenas exhibited reduced occupancy and higher detection in the wet season (Table 4). Leopards maintained consistent occupancy and detection probability between seasons, although occupancy showed very large CIs in the wet season. Cheetahs were not detected in the wet season and striped hyaenas were detected only once, consistent with their already low detection probabilities in the dry season (Table 4). In Figure 2 we present the resulting detection and occupancy coefficients and covariates from the selected models for each of the five carnivore species and season.

**TABLE 4:** Log‐transformed occupancy (Ψ) and detection (ρ) probability with 95% credible interval in the dry and wet season for the five large carnivore species detected.

	Dry season		Wet season	
	*Ψ*	*ρ*	*Ψ*	*ρ*
Lion	0.59 (0.41–0.76)	0.17 (0.10–0.25)	0.77 (0.44–0.95)	0.09 (0.05–0.13)
Spotted hyaena	0.95 (0.87–0.99)	0.05 (0.006–0.30)	0.65 (0.45–0.83)	0.33 (0.19–0.51)
Leopard	0.57 (0.41–0.75)	0.12 (0.07–0.21)	0.51 (0.14–0.93)	0.09 (0.04–0.21)
Cheetah	0.31 (0.12–0.67)	0.06 (0.03–0.13)		
Striped hyaena	0.54 (0.41–0.68)	0.10 (0.06–0.15)		

**FIGURE 3:**
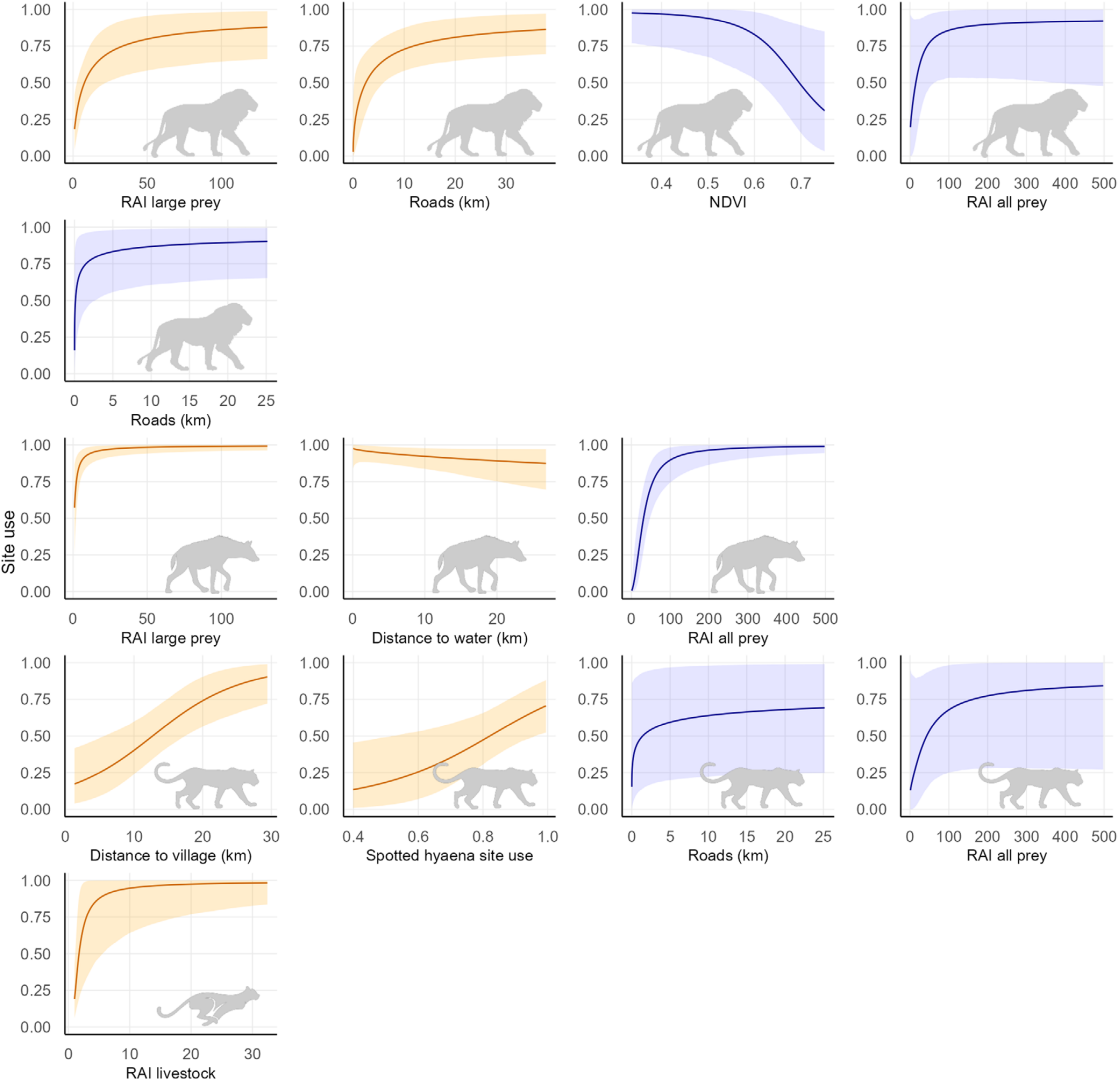
Factors influencing intensity of site use for lion, spotted hyaena, leopard and cheetah in the dry season (orange) and in the wet season (blue).

Lion intensity of site use (Figure 3) was positively associated with road density in both the dry (*β* = 1.69, 95% CI: 0.48 to 3.30) and wet season (*β* = 1.80, 95% CI: 0.40 to 3.56). It was also associated with prey abundance in both seasons: in the dry season it was positively associated with large prey abundance (*β* = 1.05, 95% CI: 0.36 to 1.96), while in the wet season there is weak evidence for positive association with abundance of all prey (*β* = 1.39, 95% CI: −0.49 to 3.49). In the wet season we note a negative effect of NDVI (*β* = −1.88, 95% CI: −3.92 to −0.16) with higher intensity of site use in areas of less productive vegetation.

Spotted hyena intensity of site use (Figure 3) in both seasons was associated with prey abundance; in the dry season it was positively associated with large prey (*β* = 1.39, 95% CI: 0.52 to 2.38), while in the wet season it was positively associated with abundance of all prey species pooled together (*β* = 2.46, 95% CI: 1.17 to 4.09). In the dry season, spotted hyaena intensity of site use also showed weak evidence for a negative association with distance to water (*β* = −0.69, 95% CI: −1.91 to 0.24).

Leopard intensity of site use (Figure 3) in the dry season was positively associated with distance from closest village (*β* = 1.02, 95% CI: 0.38 to 1.79) and spotted hyaena intensity of site use (*β* = 0.86, 95% CI: 0.19 to 1.66). While in the wet season leopard intensity of site use only showed weak evidence for positive association with park road density (*β* = 1.39, 95% CI: −0.54 to 3.59) and for all prey abundance (*β* = 1.38, 95% CI: −0.55 to 3.53).

Cheetah intensity of site use (Figure 3) was positively associated with livestock abundance in the dry season (*β* = 1.78, 95% CI: 0.51 to 3.79). None of the covariates we considered explained intensity of site use of striped hyaena in the dry season, while we did not have enough detections to model wet season intensity of site use.

## 4 Discussion

Seasonal changes in resource availability and risks trigger behavioural responses in carnivores to balance foraging necessities against the risk of human or intraguild encounters. As we expected given the strong seasonal differences in the environment in Zakouma NP, drivers of intensity of site use for each species tended to differ between the wet and dry seasons. Additionally, we had considerably fewer detections of carnivores in the wet season compared to the dry season. This might be partially due to the intrinsic limitations of camera trapping as a monitoring method in the wet season (Cusack et al. 2015); in our study most camera traps in the wet season were not accessible, and this caused the batteries to run out, the memory cards to fill up, or the grasses to grow in front of the cameras and block the view before the survey period was concluded, which led to 47% fewer sites and 75% fewer trap nights in the wet season.

Detection probability is overall low for all species and tends to be lower in the wet season. Occupancy probability shows evidence of a wet season decrease for spotted hyaenas, and presumably for cheetahs and striped hyaenas, but not for leopards and lions. In Zakouma NP, lions shift their territories and spend significantly less time inside the park in the wet season compared to the dry season (Fraticelli et al. 2025), thus we expected the wet season occupancy to be lower. Contrarily, our results show that lion site use increases in the wet season. This could be explained by the fact that, while lions spend proportionally more time outside of the park, they also increase home range size in the wet season (Fraticelli et al. 2025). Thus, while the intensity of use of areas outside of the park increases in the wet season, this does not indicate a decrease of use of the area within the park. Leopards seem to maintain similar occupancy in the park in both seasons, although the large CIs in the wet season indicate considerable uncertainty around the estimate and limit our confidence regarding seasonal differences.

Cheetah detections were low in the dry season when other species were more often detected and absent during the wet season. Shams et al. (2024) similarly did not detect any cheetah in the wet season in Zakouma NP, even though the deployment of camera traps in their study was more adapted to cheetah detection. In both studies, the number of trap nights in the wet season was relatively low, which may have contributed to the lack of detections, considering the wide ranging behaviour and low population density (0.37 individuals/100 km^2^; Shams et al. 2024). We considered a possible seasonal shift out of the park as observed in other species (Clark et al. 2023; Fraticelli et al. 2025; Labuschagne 2014), but collar data suggest that this is not a plausible explanation. Collared cheetahs did not go out of the park during the wet season in 2013 (African Parks, unpublished data) and 2023 (A. Shams, unpublished data). With only one collared individual each, we have to be cautious; but if cheetah stay within the park throughout the year, then perhaps this just shows the limits of camera trap monitoring in detecting some species in the wet season (Cusack et al. 2015). Further research with more adapted methods, such as collaring with a larger sample size, is required to gain a better understanding of wet season movement patterns of cheetah in the GZE.

Striped hyaenas showed the most drastic detection difference between dry and wet season detections, and none of the covariates included in the model had an effect on intensity of site use for this species. One site disproportionately reduced model fit, which we were unable to explain by examination of its covariate values and spatial location. Outside of Africa, striped hyaenas are positively influenced by open landscape, access to water and den availability, and show contrasting influence by anthropogenic presence as this can both provide scavenging opportunities or present a threat (Bhandari et al. 2021; Rezaei et al. 2022; Shamoon and Shapira 2019). While in our study, the presence of water influenced detection probability, we did not detect the influence of other factors on striped hyaena site use. In Africa, little is known about striped hyaenas (Strampelli et al. 2022; Wilkinson, Dheer, et al. 2024) and our results show a gap in understanding and a potential risk for species conservation in southern Chad. Further research focused on understanding wet season movement, potentially with satellite collars whose data collection is not limited by seasonal constraints or geographic distribution, is recommended for this species.

The wet season limitations of our study prevent us from making strong assumptions on the reasons behind the seasonal differences in occupancy of large carnivores in Zakouma NP. Interpreting these results with the knowledge that collared lions move outside of the park (Fraticelli et al. 2025) and that human‐wildlife conflict for lions and leopards increases in the wet season (Fraticelli et al. 2026) might suggest that large carnivores exit the park to some extent in the wet season when anthropogenic presence is lower, potentially in search of prey and to avoid the floods. But this difference could also be due to animals being forced into smaller areas due to flooding which were not appropriately covered by the camera traps. In Namibia, Verschueren et al. (2021) found little seasonal differences in carnivore occupancy, but the landscape had permanent and artificial water points and a non‐migratory prey base. In a more similar landscape in southern Kenya, seasonal changes in human and livestock density were a key driver of carnivore occupancy (Schuette, Wagner, et al. 2013).

### 4.1 Covariate Associations

Detection of the three largest species was higher on the roads, which are known to facilitate communication, foraging and movement of carnivores (Hill et al. 2021). Cheetah, on the other hand, are well known to have higher detections at marking sites and vantage points compared to roads (Drouilly et al. 2025, 2019), and this was also noted in another camera trap study in Zakouma NP in 2023, where 67% of detections of cheetah were at marking sites (Shams et al. 2024).

Large prey abundance increased the intensity of site use of lions and spotted hyaenas in the dry season. These findings are in line with published literature on prey preference of lions (Clements et al. 2014; Fraticelli et al. 2024; Hayward and Kerley 2005). Preferred weight range of prey for spotted hyaena falls in the medium category, but they are generalists and feed on a wide range of available prey (Clements et al. 2014), and in Hwange NP in Zimbabwe they were found to increase consumption and preference of large prey in the presence of high lion abundance (Périquet, Fritz, and Revilla 2015; Périquet, Valeix, et al. 2015). Collar data shows that in the wet season, some large and medium prey species migrate out of Zakouma NP and all prey is more dispersed (African Parks, unpublished data; Clark et al. 2023; Poilecot 2006). This likely explains why the intensity of site use of lions, leopards and spotted hyaenas in the wet season is not linked to size‐specific prey abundance, but rather to total prey abundance.

Water did not have a strong direct effect on the intensity of site use for any species in either season at site‐scale. Carnivores are not water dependent but they tend to aggregate around water sources as these attract prey species (Valeix et al. 2010), but not all waterholes are equally visited by predators (Dejeante et al. 2025). For example, in Nakuru county in Kenya spotted hyaenas avoided rivers but were attracted to lakes (Wilkinson, Xu, et al. 2024). In Zakouma NP, surface water in the dry season is restricted to a few large permanent pans and scattered ponds in the riverbeds. In our analysis we did not distinguish between the type or extent of water available, while this might be an important factor for prey abundance and thus link to predator habitat use. In the wet season, water is widespread and unlikely to act as an attractant for prey or predator, but rather we expected an avoidance of areas with higher surface water, but our results did not show this. The placement of cameras to avoid flood damage thus avoiding areas with the higher water presence might have hidden this pattern at the study scale, but the lower detections of predators throughout the park might also indicate an avoidance of floods at a larger scale.

In the wet season, lions avoided areas with a high Normalised Difference Vegetation Index (NDVI), corresponding to highly productive areas with dense, green vegetation and greater plant biomass. Similar patterns have been reported elsewhere: in northern Botswana, vegetation productivity was negatively associated with lion occupancy (Rich et al. 2017), and in the Kavango‐Zambezi Transfrontier Conservation Area lions selected areas with low to moderate positive NDVI and avoided areas with high NDVI (Bauer et al. 2025). High productivity promotes woody vegetation, reducing grass availability and subsequently the presence of grassland‐dependent prey. However, responses vary by landscape context; for example, in human‐impacted systems such as Ngorongoro in Tanzania, lions selected denser vegetation for concealment and to reduce human encounters (Jansson et al. 2024).

Distance from park boundary was not found to influence intensity of site use in any of the models, indicating that none of the species avoid areas closer to park boundaries in either season. In the Serengeti ecosystem, research showed a slight edge effect on lion probability of use within the core protected area in the wet season, and a significant difference in site use in the dry season, but only at the border with buffer areas with lower protection, when human and livestock incursions increase (Schooler et al. 2022). Our results show no edge effect even though Zakouma NP has several villages in the immediate vicinity, and nomad transhumants settle along the borders during the dry season. A transhumant lifestyle means anthropogenic effects are not constant in space and time, and thus wildlife is likely to adapt within season on a finer scale of avoidance not detected by our study. In the wet season, the absence of transhumant pastoralists, who move north with the rains, decreases the anthropogenic pressure outside of the park, allowing for increased movement of wildlife between the park and the community areas (Clark et al. 2023; Fraticelli et al. 2025; Labuschagne 2014).

While there was no evidence of park boundary leading to an edge effect for any species, leopards showed an avoidance of permanent villages in the dry season, but not in the wet season. This is in accordance with the results of enquiries on the perceived human wildlife conflict in the area that show that villagers report more conflict with leopards in the wet season compared to the dry season (Fraticelli et al. 2026). On the other hand, cheetah probability of site use increased with the abundance of livestock in the dry season. This could be due to the predation opportunity that small stock such as goats and sheep offer for cheetah, but predation by cheetah is not commonly reported around Zakouma NP (African Parks, unpublished data) and analysis on 2023 data in the area shows that they have spatial overlap but no temporal overlap with livestock (Shams 2025).

We did not find any spatial avoidance of dominant predators by subordinate predators; instead, we found that leopard intensity of site use increased with that of spotted hyaenas. An increase of occupancy probability of subordinate carnivores in the presence of dominant predators was also noted in Hwange NP in Zimbabwe (Morin et al. 2024), while other studies have found sex‐specific behavioural adaptation of leopards in the presence of spotted hyaenas, with the latter avoiding adult male leopards (Davis et al. 2021; Havmøller et al. 2024). Interactions between sympatric large carnivores are varied and can range from competitive interactions, such as kleptoparasitism (Balme, Miller, et al. 2017; Périquet, Fritz, and Revilla 2015; Périquet, Valeix, et al. 2015) and intraguild predation (Curveira‐Santos et al. 2022), to facilitation, such as scavenging opportunities (Say‐Sallaz et al. 2025), and are context dependent (Greco et al. 2021; Haswell et al. 2017). Coexistence between lion and spotted hyaena is improved by increased presence of prey, and their density is not driven by competitor presence (Périquet, Fritz, and Revilla 2015; Périquet, Valeix, et al. 2015; Périquet et al. 2025). Similarly, with adequate availability of medium to large prey, lion presence does not suppress leopard population nor limit their distribution (Balme, Pitman, et al. 2017; Bashant et al. 2020; Rafiq et al. 2020), which is likely the case in Zakouma NP where lions mainly prey on large prey (Fraticelli et al. 2024) and leopards do not avoid lion areas. Several studies found no effect of lions on leopard numbers, distribution or diel activity patterns (Balme, Pitman, et al. 2017; Bashant et al. 2020; Miller et al. 2018). Leopard behaviour of hoisting kills on trees has been found to be an effective way to avoid kleptoparasitism by spotted hyaenas (Balme, Miller, et al. 2017) and promote coexistence. For cheetah, studies found temporal avoidance and not spatial avoidance of dominant predators (Cornhill et al. 2023; Dröge et al. 2017; Hayward and Slotow 2009), which is not an aspect we explored in this study.

### 4.2 Conservation Implications

Zakouma NP acts as a refuge within the ecosystem in the dry season, when anthropogenic pressure is higher in the community lands due to the presence of transhumant pastoralists. Prey availability resulted as one of the main drivers of intensity of site use for most species in both seasons, and there is no evidence of intraguild competition between the five large carnivore species detected. In Botswana, in the dry season resource availability was more important than intraguild interactions in shaping large carnivore occupancy, while in the wet season the distribution of leopard, spotted hyaena, and wild dogs was best predicted by intraguild competition (Rich et al. 2017).

In the wet season, the decrease in anthropogenic presence in the wider landscape allows for a range shift outside of the park for many species, at a time when prey base decreases inside the park (Fraticelli et al. 2024) and floods render parts of the park unsuitable. While this explanation is supported by collar data for lions, there is more uncertainty for other species. In southern Kenya, seasonal changes in human land use with shifting densities of humans and livestock were a key driver of carnivore occupancy (Schuette, Wagner, et al. 2013) and promoted the coexistence of lions and livestock at relatively high densities (Schuette, Creel, and Christianson 2013). In the GZE, the seasonal movement of pastoralists is essential to allow large carnivores to persist in the landscape, and disruptions to these seasonal dynamics can increase human‐wildlife conflict and impact carnivore populations (Ayantunde et al. 2014; McGuirk and Nunn 2024).

Our study, specifically the wet season component, was limited by constraints on accessibility. Nonetheless, our results contribute important information to the understanding of the wet season dynamics in Zakouma NP, which can help guide management decisions. This study was also limited to the extent of the national park and did not include any cameras outside of the park in the community areas. The deployment of cameras on the border roads, the encroachment of livestock within the park and the presence of villages inside park boundaries and in the immediate vicinity allowed us to explore how these anthropogenic factors affect carnivore habitat use in the core protected area. The transition period between the dry and wet seasons was not included in this analysis, but may provide interesting insights into how large carnivores respond to rapidly changing resource distributions, flooding dynamics and human activity patterns. Explicitly targeting this period could improve understanding of seasonal movement strategies and fine‐scale temporal responses to pressures within the GZE. Future studies should also expand to the community areas to better understand if the decrease of detection and occupancy of Zakouma NP's carnivores in the wet season is reflected as a contemporary increase in the community lands, and if drivers of site use here might be different compared to core protected areas (Jansson et al. 2024; Oriol‐Cotterill, Macdonald, et al. 2015; Oriol‐Cotterill, Valeix, et al. 2015; Wilkinson, Xu, et al. 2024).

## Author Contributions

**Chiara Fraticelli:** conceptualization (lead), data curation (lead), formal analysis (lead), investigation (lead), methodology (lead), project administration (lead), resources (lead), visualization (lead), writing – original draft (lead), writing – review and editing (equal). **Ali Shams:** writing – review and editing (equal). **Stijn Verschueren:** writing – review and editing (equal). **Ousmane Ourde:** data curation (equal), investigation (equal), writing – review and editing (equal). **Baudin Fekoua:** data curation (supporting), investigation (equal), writing – review and editing (equal). **Abdoulaye Abakar Zayed:** data curation (supporting), investigation (equal), writing – review and editing (supporting). **Herwig Leirs:** conceptualization (supporting), supervision (supporting), writing – review and editing (supporting). **Hans Bauer:** conceptualization (supporting), supervision (supporting), writing – original draft (supporting), writing – review and editing (equal).

## Funding

This work was supported by Wildlife Conservation Network's Lion Recovery Fund, AN‐PA‐01, CH‐AP‐03‐LRF, CH‐AP‐02‐LRF.

## Conflicts of Interest

The authors declare no conflicts of interest.

## Supporting information


**Table S1:** Model selection for occupancy (*Ψ*) and detection (*ρ*) probabilities of five large carnivores in the dry and wet season. *k* represents the number of estimated parameters; *p* is the Bayesian *p*‐value (*p* sites: across sites; and *p* rep: across replicates) only calculated for models with ΔWAIC < 2. For covariate definitions, please refer to Table 1.
**Figure S1:** Freeman‐Tukey graphs and Bayesian *p*‐values for top models for each species and season.

## Data Availability

The data that support the findings of this study are openly available in Dryad at https://doi.org/10.5061/dryad.j9kd51cvc.
