# Supplementary material for: Seasonal Dynamics of Resource Availability and Human Presence Shape Habitat Use of Large Carnivores in Chad
Source: Ecol Evol. 2026 Jul 13;16(7):e73957. doi: 10.1002/ece3.73957 (PMC13361059; doi:10.1002/ece3.73957)

**Appendix**

Table S1: Model selection for occupancy (Ψ) and detection (ρ) probabilities of five large carnivores in the dry and wet season. k represents the number of estimated parameters; p is the Bayesian p-value (p sites: across sites; and p rep: across replicates) only calculated for models with ΔWAIC<2. For covariate definitions, please refer to Table 1.

| Dry season |  |  |  |  |  | Wet season |  |  |  |  |  |
| --- | --- | --- | --- | --- | --- | --- | --- | --- | --- | --- | --- |
| Models | k | WAIC | ΔWAIC | p sites | p rep | Models | k | WAIC | ΔWAIC | p sites | p rep |
| Lion |  |  |  |  |  |  |  |  |  |  |  |
| **Ψ(Pla+Road) ρ(locR+so)** | **6** | **781.81** | **0.00** | **0.12** | **0.50** | **Ψ(Pall+Road+NDVI) ρ(.)** | **5** | **162.88** | **0.00** | **0.42** | **0.25** |
| Ψ(Pla+Road+Wdry) ρ(locR+so) | 7 | 784.14 | 2.33 |  |  | Ψ(Road+NDVI) ρ(.) | 4 | 164.97 | 2.08 |  |  |
| Ψ(Road) ρ(locR+so) | 5 | 788.83 | 7.03 |  |  | Ψ(Pall+Road) ρ(.) | 4 | 168.88 | 5.99 |  |  |
| Ψ(Pla) ρ(locR+so) | 5 | 789.85 | 8.05 |  |  | Ψ(Pall+NDVI) ρ(.) | 4 | 170.17 | 7.29 |  |  |
| Ψ(Road+Wdry) ρ(locR+so) | 6 | 789.93 | 8.12 |  |  | Ψ(Road) ρ(.) | 3 | 170.99 | 8.11 |  |  |
| Ψ(Pla+Wdry) ρ(locR+so) | 6 | 791.64 | 9.83 |  |  | Ψ(NDVI) ρ(.) | 3 | 172.09 | 9.21 |  |  |
| Ψ(Wdry) ρ(locR+so) | 5 | 800.64 | 18.83 |  |  | Ψ(Pall) ρ(.) | 3 | 172.63 | 9.75 |  |  |
| Spotted hyaena |  |  |  |  |  |  |  |  |  |  |  |
| **Ψ(Pla+Wdry) ρ(locR+so+eff)** | **7** | **1723.48** | **0.00** | **0.00** | **0.61** | **Ψ(Pall) ρ(so+locR)** | **5** | **310.08** | **0.00** | **0.36** | **0.69** |
| Ψ(Pla) ρ(locR+so+eff) | 6 | 1723.84 | 0.37 | 0.00 | 0.61 | Ψ(Pall+lion) ρ(so+locR) | 6 | 311.65 | 1.57 | 0.36 | 0.69 |
| Ψ(Pla+Wdry+lion) ρ(locR+so+eff) | 8 | 1725.05 | 1.58 | 0.00 | 0.62 | Ψ(lion) ρ(so+locR) | 5 | 324.67 | 14.59 |  |  |
| Ψ(Pla+lion) ρ(locR+so+eff) | 7 | 1725.51 | 2.04 |  |  |  |  |  |  |  |  |
| Ψ(Wdry+lion) ρ(locR+so+eff) | 7 | 1727.73 | 4.25 |  |  |  |  |  |  |  |  |
| Ψ(lion) ρ(locR+so+eff) | 6 | 1729.31 | 5.83 |  |  |  |  |  |  |  |  |
| Ψ(Wdry) ρ(locR+so+eff) | 6 | 1733.87 | 10.40 |  |  |  |  |  |  |  |  |
| Leopard |  |  |  |  |  | (10-day sampling occasion) | | | | | |
| **Ψ(Villa+sphy) ρ(so+locR)** | **6** | **642.07** | **0.00** | **0.12** | **0.70** | **Ψ(Pall+Road) ρ(.)** | **4** | **73.39** | **0.00** | **0.46** | **0.62** |
| Ψ(Villa+Park+sphy) ρ(so+locR) | 7 | 643.85 | 1.78 | 0.10 | 0.69 | Ψ(Road) ρ(.) | 3 | 74.60 | 1.21 | 0.41 | 0.62 |
| Ψ(Park+sphy) ρ(so+locR) | 6 | 644.35 | 2.27 |  |  | Ψ(Pall) ρ(.) | 3 | 75.38 | 1.99 | 0.44 | 0.61 |
| Ψ(Villa) ρ(so+locR) | 5 | 648.08 | 6.01 |  |  |  |  |  |  |  |  |
| Ψ(Villa+Park) ρ(so+locR) | 6 | 649.28 | 7.21 |  |  |  |  |  |  |  |  |
| Ψ(Park) ρ(so+locR) | 5 | 649.78 | 7.70 |  |  |  |  |  |  |  |  |
| Ψ(sphy) ρ(so+locR) | 5 | 649.92 | 7.85 |  |  |  |  |  |  |  |  |
| Cheetah (10-day sampling occasion) | | | | | |  |  |  |  |  |  |
| **Ψ(stock) ρ(.)** | **3** | **133.76** | **0.00** | **0.64** | **0.78** |  |  |  |  |  |  |
| Ψ(.) ρ(.) | 2 | 143.11 | 9.35 |  |  |  |  |  |  |  |  |
| Striped hyaena (excluding outlier) | | | | | |  |  |  |  |  |  |
| **Ψ(.) ρ(so+locW)** | **4** | **658.57** | **0.00** | **0.19** | **0.36** |  |  |  |  |  |  |

Figure S1: Freeman-Tukey graphs and Bayesian p-values for top models for each species and season.

Lion, dry season: Ψ(Pla+Road) ρ(locR+so)


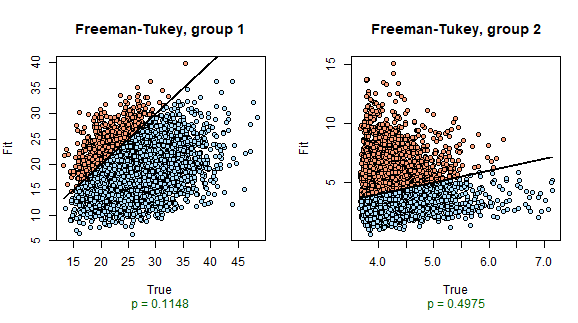


Spotted hyaena, dry season: Ψ(Pla+Wdry) ρ(locR+so+eff)


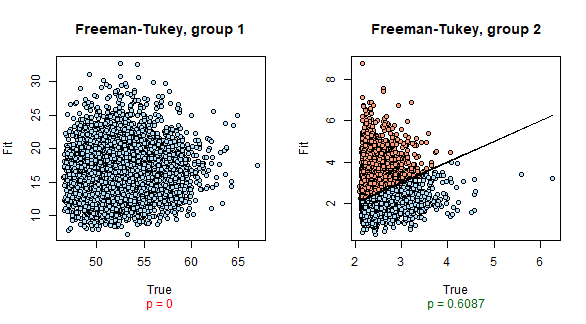


Leopard, dry season: Ψ(Villa+sphy) ρ(so+locR)


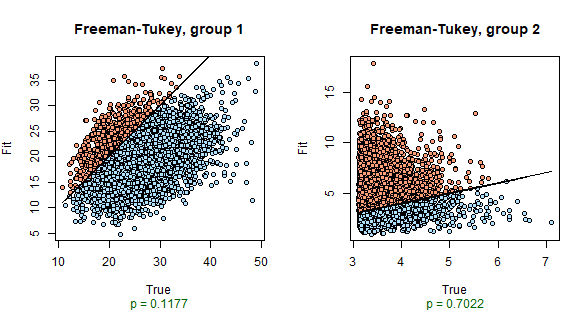


Cheetah, dry season (5-day sampling occasion): Ψ(stock) ρ(.)


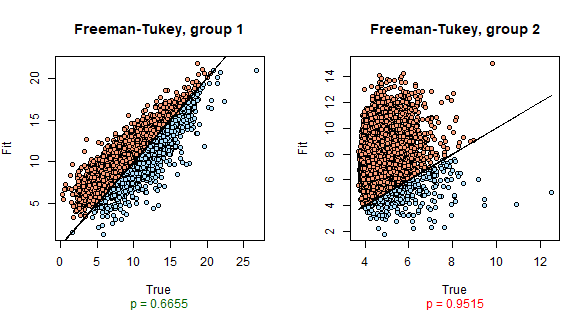


Cheetah, dry season (10-day sampling occasion): Ψ(stock) ρ(.)


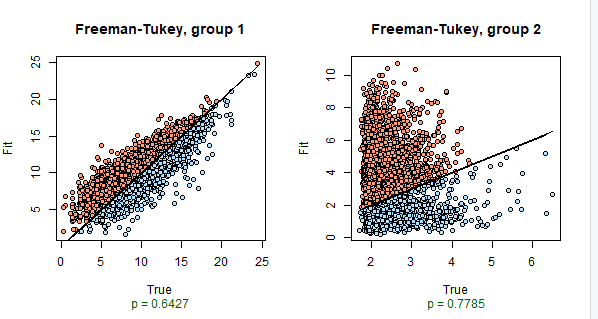


Striped hyaena, dry season: Ψ(.) ρ(so+locR+locW)


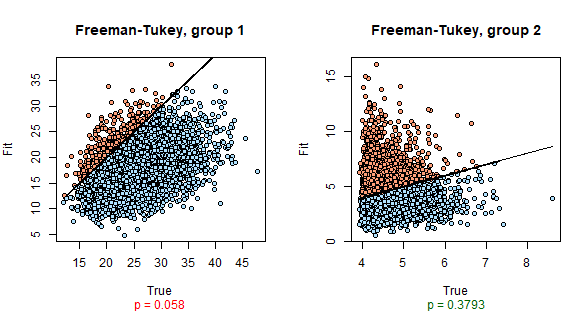


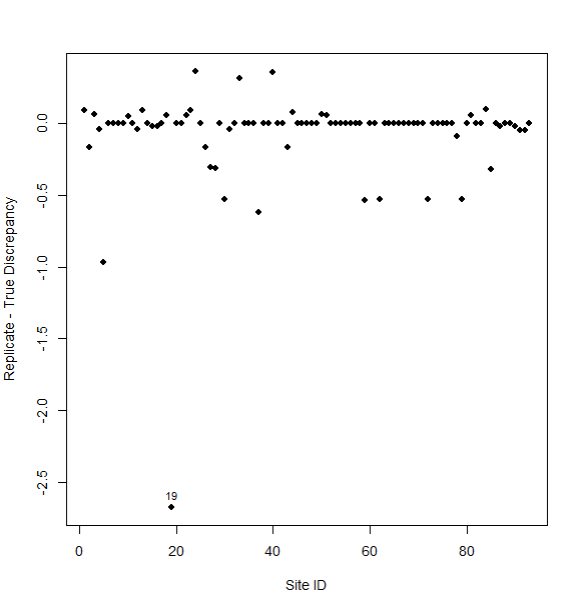


Striped hyaena, dry season (excluding outlier site 19): Ψ(.) ρ(so+locW)


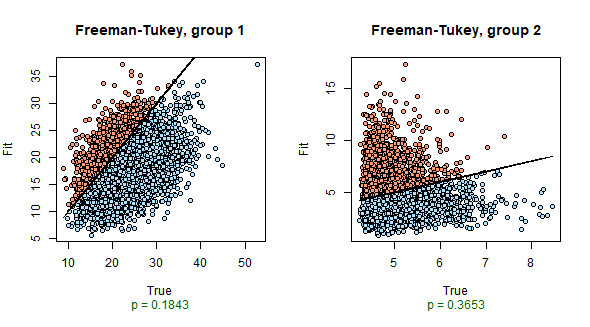


Lion, wet season: Ψ(Pall+Road+NDVI) ρ(.)


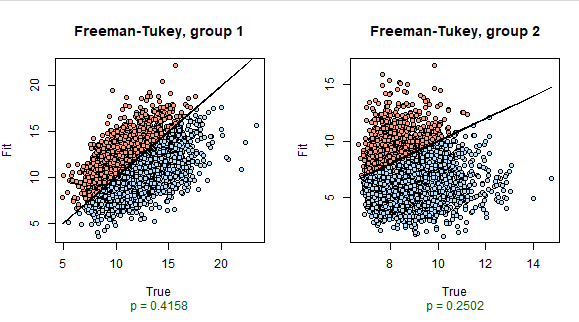


Spotted hyaena, wet season: Ψ(Pall) ρ(so+locR)


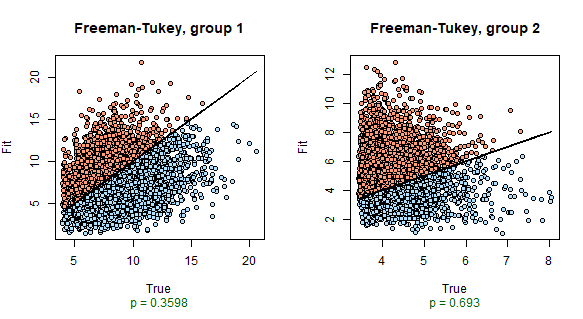


Leopard, wet season (5-day sampling occasion): Ψ(Pall+Road) ρ(.)


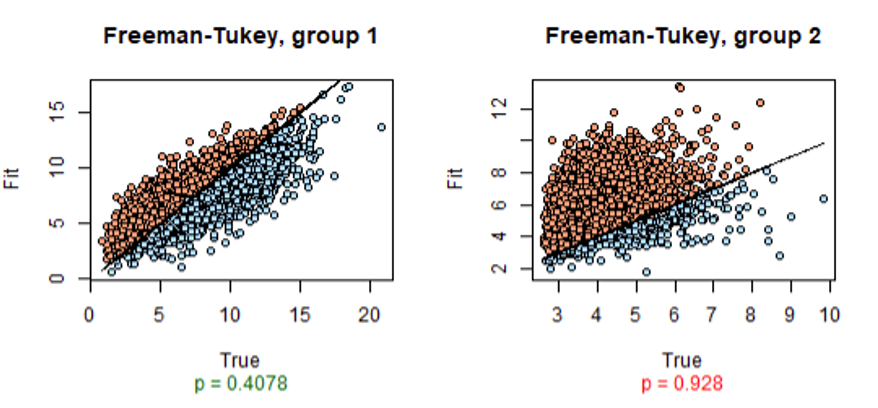


Leopard, wet season (10-day sampling occasion): Ψ(Pall+Road) ρ(.)


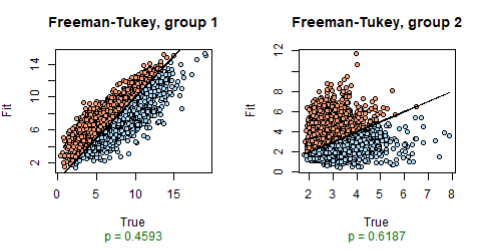

Supplement: Supplementary file 1 — Table S1: Model selection for occupancy (Ψ) and detection (ρ) probabilities of five large carnivores in the dry and wet season. k represents the number of estimated parameters; p is the Bayesian p‐value (p sites: across sites; and p rep: across replicates) only calculated for models with ΔWAIC < 2. For covariate definitions, please refer to Table 1. Figure S1: Freeman‐Tukey graphs and Bayesian p‐values for top models for each species and season. [file ECE3-16-e73957-s001.docx]
